# ^56^Fe ion irradiation enhances angiogenesis and other inter-cellular determinants of carcinogenesis risk

**DOI:** 10.1093/jrr/rrt180

**Published:** 2014-03

**Authors:** Swati Girdhani, Clare Lamont, Michael Peluso, Maoyun Sun, Lynn Hlatky

**Affiliations:** Center of Cancer Systems Biology, GRI, Tufts University School of Medicine, 736 Cambridge Street, SEMC-CBR112, Boston, MA 02135, USA

## Abstract

In the assessment of radiogenic cancer risk from space flight, it is imperative to consider effects not only on the creation of cancer cells (initiation) but also on cell–cell interactions that play an important and often decisive role in the promotion and progression phases. Autopsy results confirm that most adults carry fully malignant tumors that are held in check at a small size and will never become symptomatic [ 1, 2]. This introduces the possibility that cosmic radiation may significantly influence cancer risk through alteration of the bottleneck inter-tissue interactions responsible for maintaining this dormant state. One such bottleneck is the growth limitation imposed by the failure of the tumor to induce blood vessels (angiogenesis). Other deciding events are the ability of a tumor to proliferate and invade. We have previously shown that proton radiation, the most prevalent radiation in space, has a suppressive effect on all three of these functional responses. It down-regulates angiogenic genes like VEGF and HIF-1α and impairs cell invasion and tumor growth [ 3]. We decided to test these responses after ^56^Fe irradiation, an HZE radiation type present in the cosmic environment with presumably high carcinogenic potential [ 4].

Human microvascular endothelial cells (HMVEC) and normal human dermal fibroblast (NHDF) cells were irradiated with different doses of ^56^Fe ion radiation (1 GeV/n) at Brookhaven National Laboratory and RNA was extracted 6 h later. Genomic-wide array analysis was done on the isolated RNA through the Agilent Platform. It was observed that several pro-angiogenic genes like VEGF, IL-6 and HIF-1α were significantly up-regulated after treatment with ^56^Fe ion radiation (Fig. 1). These results were also confirmed at the mRNA and protein levels with the human and murine lung cancer lines, A549 and LLC, respectively. Additional verification of modulation of these key genes was also observed when lungs of C57BL/6 mice treated with ^56^Fe ion radiation showed an increase in VEGF and MMP9 mRNA and protein expression 6 h post-irradiation (Fig. 2). Cell invasion was shown to be increased by ^56^Fe ion radiation in various cell types, including fibroblast, tumor and endothelial progenitor cells. ^56^Fe ion irradiation also modulated functional processes crucial to angiogenesis. It enhanced the ability of untargeted (bystander) endothelial cells to invade and proliferate in response to factors produced by targeted fibroblast or cancer cells *in vitro*. Results also carry over to *in vivo*. C57BL/6 mice exposed to whole-body irradiation with 0.2 Gy dose of ^56^Fe and injected subcutaneously with LLC tumor cells showed a significant augmentation in tumor growth and growth rate in the irradiated group. Additionally, nude mice exposed to whole-body ^56^Fe radiation and injected intravenously with A549 cancer cells 3 h post-irradiation demonstrated a significant enhancement in lung colonization capacity when compared with the sham-irradiated control mice injected.

These results together suggest cell and tissue-level responses to ^56^Fe irradiation may act to overcome major cancer progression-level bottlenecks including those related to angiogenesis, cell proliferation and invasion. This is of significant concern for cancer risk estimations pertinent to NASA as achieving these cancer hallmark processes can make the difference between a radiation-induced cancer cell progressing to a clinically detectable cancer in astronauts or not. In conclusion, we demonstrate a strong radiation quality dependence for space radiation carcinogenesis risk manifested through influences on intercellular interactions in the progression phase of carcinogenesis.

**Fig. 1.**
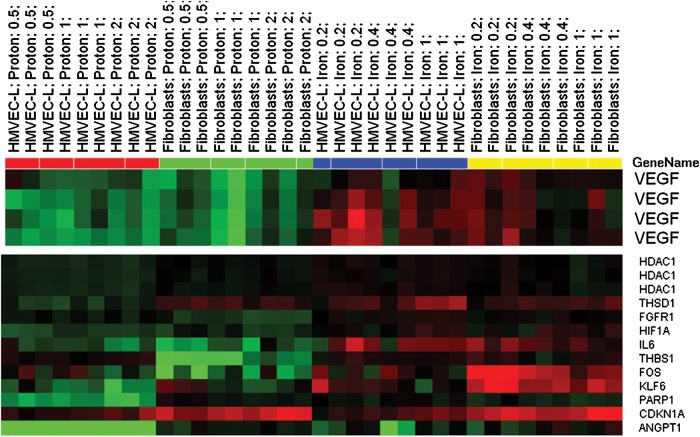
Heatmaps of selected differentially regulated major angiogenesis genes after proton and ^56^Fe ion radiation in HMVECs and NHDF. Cells were treated with either 0, 0.5, 1 or 2 Gy of proton radiation or 0, 0.2, 0.4 or 1 Gy of ^56^Fe ion dose. Among the major regulated genes were VEGF, HIF-1A and IL-6; they were down-regulated by proton radiation and up-regulated by iron radiation.

**Fig. 2.**
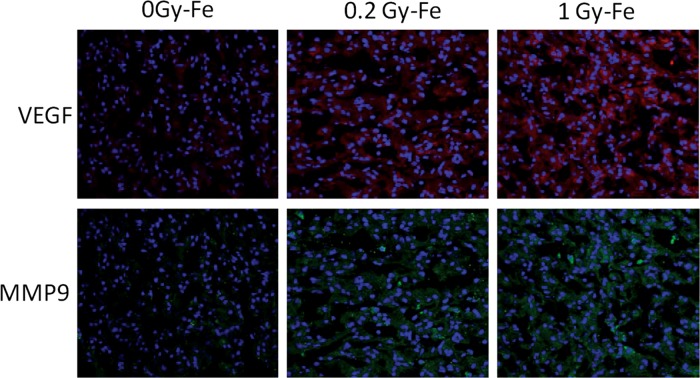
Immunofluorescence images of lungs of C57BL/6 mice treated with 0, 0.2 or 1 Gy of ^56^Fe ion dose and stained 6 h later. Pro-angiogenic factors VEGF and MMP9 were increased in mice that received the ^56^Fe ion treatment.

Heatmaps of selected differentially regulated major angiogenesis genes after proton and ^56^Fe ion radiation in HMVECs and NHDF. Cells were treated with either 0, 0.5, 1 or 2 Gy of proton radiation or 0, 0.2, 0.4 or 1 Gy of ^56^Fe ion dose. Among the major regulated genes were VEGF, HIF-1A and IL-6; they were down-regulated by proton radiation and up-regulated by iron radiation.

Immunofluorescence images of lungs of C57BL/6 mice treated with 0, 0.2 or 1 Gy of ^56^Fe ion dose and stained 6 h later. Pro-angiogenic factors VEGF and MMP9 were increased in mice that received the ^56^Fe ion treatment.

## References

[1] Black WC, Welch HG (1993). Advances in diagnostic imaging and overestimations of disease prevalence and the benefits of therapy. N Engl J Med.

[2] Hahnfeldt P (2013). The host support niche as a control point for tumor dormancy: implications for tumor development and beyond. Adv Exp Med Biol.

[3] Girdhani S, Lamont C, Hahnfeldt P (2012). Proton irradiation suppresses angiogenic genes and impairs cell invasion and tumor growth. Radiat Res.

[4] Durante M, Cucinotta FA (2008). Heavy ion carcinogenesis and human space exploration. Nat Rev Cancer.

